# THE USE OF ADHESIVE RADIOPAQUE GRIDS IN GENICULAR NERVE BLOCK BY RADIOSCOPY

**DOI:** 10.1590/1413-785220233103e266060

**Published:** 2023-07-17

**Authors:** DANIEL PEIXOTO LEAL, MATHEUS GARCIA LOPES MERINO, MUHAMAD MUSTAFA ATIEH, VITOR HENRIQUE CAMPOY GUEDES, JOSE RICARDO PECORA, CAMILO PARTEZANI HELITO

**Affiliations:** 1 Universidade de São Paulo, Faculdade de Medicina, Hospital das Clínicas, Instituto de Ortopedia e Traumatologia IOT HCFMUSP, Departamento de Ortopedia, Grupo de Joelho, São Paulo, SP, Brazil.; 2 Universidade de São Paulo, Faculdade de Medicina, Hospital das Clínicas, Instituto de Ortopedia e Traumatologia IOT HCFMUSP, Departamento de Ortopedia, São Paulo, SP, Brazil.

**Keywords:** Nerve Block, Orthopedic Surgeons, Peripheral Nerves, Bloqueio Nervoso, Cirurgiões Ortopédicos, Nervos Periféricos

## Abstract

The performance of genicular nerve block requires an imaging method to guide the procedure. Radioscopy has the disadvantage of being radiation dependent.

**Objective:**

To assess whether the use of adhesive radiopaque grids reduce radiation exposure in these cases.

**Methods:**

This is a cross-sectional study conducted with 23 orthopedists in which needles were positioned in a model with and without the use of adhesive radiopaque grids. The number of fluoroscopy shots necessary for proper positioning in three points (superior lateral, superior medial, and inferior medial) were registered.

**Results:**

A statistical difference was observed in the three blocking points studied. The number of radioscopies required for these three points were 12.1 ± 2.5 in the group without grid and 5.0 ± 1.8 in the group with grid. The superior medial point presented the greatest numerical difference and the inferior medial point the smallest.

**Conclusion:**

The use of adhesive radiopaque grids led to a statistically significant reduction in the number of radioscopies/fluoroscopies required to perform the genicular block. The use of this device increases the safety of the physician and patient by reducing radiation exposure in this procedure. ***Level of Evidence III, Level of Evidence II, Random Clinical Trial.***

## RESUMO

Para a realização do bloqueio de nervos geniculares é necessário guiar o procedimento por um método de imagem. A radioscopia possui a desvantagem de ser dependente de radiação.

**Objetivo:**

Avaliar se o uso de máscaras localizadoras diminui a exposição à radiação nesses casos.

**Métodos:**

Estudo transversal realizado com 23 ortopedistas, que realizaram o posicionamento de agulhas em um modelo com e sem o uso da máscara localizadora. Foi registrado o número de escopias necessárias para o posicionamento adequado em três pontos: superior lateral, superior medial e inferior medial.

**Resultados:**

Foi observada diferença estatística nos três pontos de bloqueio analisados. O número de radioscopias necessárias nos três pontos somados foi de 12,1 ± 2,5, no grupo sem máscara, e 5,0 ± 1,8, no grupo com máscara. O ponto superior medial foi o que apresentou a maior diferença numérica, e o inferior medial a menor.

**Conclusão:**

O uso da máscara localizadora reduziu de forma estatisticamente significativa o número de escopias necessárias para a realização do bloqueio genicular. O uso desse dispositivo aumenta a segurança do médico e do paciente por diminuir a exposição à radioscopia nesse procediment. **Nível de Evidência III, Ensaio clínico randomizado aberto - Nível de recomendação B - nível de evidência 2b.**

## INTRODUCTION

Knee osteoarthritis is a disease that affects approximately 1/3 of the global population over 65 years old, causing pain and impairment in quality of life. For the treatment of gonarthrosis, there are options such as physiotherapy, orthoses, acupuncture, pain relievers, joint injections, among others. If conservative treatment fails, total knee arthroplasty (TKA) is chosen.^1^ Choi et al., ^(^ ^2^ in 2011, introduced the use of genicular nerve block (pharmacological or radiofrequency) as an alternative treatment for chronic knee pain. Since then, in patients with refractory pain despite conservative treatment and who are not eligible for TKA due to comorbidities, the use of genicular nerve block has been shown to be a good option. In addition, about 15 to 30% of patients who undergo TKA continue to experience pain and functional limitation in the knee, and the use of genicular nerve block has been shown to be effective in treating these residual symptoms. ^(^ ^1^ ^),(^ ^3^ Other applications of genicular nerve block include its use in patients with rheumatoid arthritis as a method to aid in the modulation of the inflammatory process, ^(^ ^4^ and in combination with intra-articular injection (corticosteroid or viscosupplementation) to enhance its analgesic effects. ^(^ ^5^

Genicular nerve block, whether pharmacological or by radiofrequency, is performed on three nerve trunks around the knee: the superior lateral, superior medial, and inferior medial genicular nerves. To perform the procedure, needles must be positioned close to these nerve trunks for medication infiltration or neurotomy. Needles can be positioned in three ways: via anatomical landmarks, which is considered a fairly imprecise method and is falling out of use; guided by ultrasound or fluoroscopy. The ultrasound-guided (USG) technique is a highly precise method for guiding the procedure, as the genicular nerve can be accurately identified, and it has the advantage of not using radiation. However, it is an examiner-dependent method that requires the availability of ultrasound equipment, which is not the reality of many medical institutions in our country. The fluoroscopy-guided method has been shown to be as effective as the ultrasound-guided method in improving pain and functionality scores in comparative studies, with the disadvantage of being a radiation-dependent method. ^(^ ^3^ ^),(^ ^6^

In our country, due to the wide availability of fluoroscopy equipment and being a simpler and examiner independent method, the fluoroscopic method is the most commonly used for genicular nerve block. A theoretical alternative to minimize radiation exposure for both the physician and the patient is the use of adhesive radiopaque grids to guide needle positioning, aiming to use a lower number of fluoroscopy scans for proper needle placement. However, studies showing objective superiority in the use of these grids to decrease radiation exposure are scarce. The published studies on guiding needles in spine procedures using grids have shown potential benefits of this method. ^(^ ^7^ ^),(^ ^8^

Thus, this study aimed to evaluate if the use of adhesive radiopaque grids significantly reduce the number of scopies and, consequently, radiation exposure in performing genicular blockade for the treatment of chronic knee pain. As a hypothesis of the study, we assume that the use of the grid reduces the number of attempts for the proper positioning of needles in the three points (superolateral, superomedial, and inferomedial) for performing knee blocks.

## METHODS

**Figure 1:**
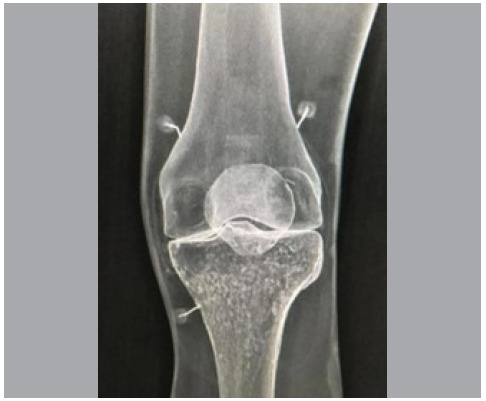
Fluoroscopy image of the anatomical model used in the study demonstrating the proper positioning of genicular block needles at the superomedial, superolateral, and inferomedial points.

For the execution of this study, a knee model was used and placed on a fluoroscopy device with a transparent table. Orthopedic surgeons who had already received instruction and training on how to perform genicular nerve blocks were instructed to position the needles on the knee model using fluoroscopy in the anteroposterior incidence. All participants signed the informed consent form to participate in the study processes and the article was approved in the protocol of the Ethics and Research Committee under the protocol number 5,920,389. The points chosen for needle placement were based on the study by Sari et al. ^3^ for blocking the superior lateral, superior medial, and inferior medial genicular nerves (Figure 1). These points were chosen since they are the most commonly used for this type of procedure. ^(^ ^3^

Each surgeon was instructed to position the needles without using a grid and with the use of a grid (X-GRID radiopaque adhesive grid, Target Tape, Canada). The initial positioning with or without the grid was randomly chosen to minimize its influence on the final results. The number of attempts made to achieve the correct positioning at each point was quantified. The positioning without the grid was based only on anatomical parameters and on sequential fluoroscopy images taken during the procedure.

**Figure 2:**
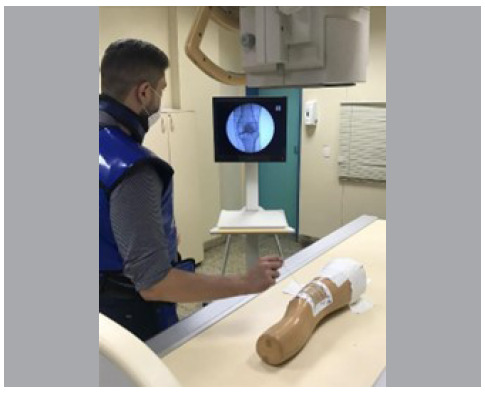
Orthopedist positioning the genicular block needles under fluoroscopy on the previously positioned model. It is possible to observe the adhesive radiopaque grid used in the study.

The knee model was positioned on the transparent table and fixed in the true anteroposterior position. The surgeon was not allowed to modify the position of the model. After attempts by the same surgeon, the model was repositioned if it had changed position (Figure 2).

A pilot study was conducted with five surgeons at the superior lateral genicular point, and the average number of attempts for correct positioning was 4.2 ± 0.8 attempts. Thus, considering a decrease of at least one attempt with the use of the grid, the sample was estimated for a power of 80% and p < 0.05 with a minimum of 14 participants.

The numerical variables were described as mean and standard deviation. Student’s t-test or Mann-Whitney U test were used to compare numerical variables between groups according to the normality of the variables.

P values < 0.05 were considered significant. The SPSS 22 (IBM Corp., NY, USA) statistical software was used.

## RESULTS

A total of 23 orthopedists were selected for the study. The means for each point performed without the grid were 5.4 ± 1.5; 3.6 ± 1.5; and 3.0 ± 1.3 for the superior medial, superior lateral, and inferior medial points, respectively. With the use of the grid, the means were 1.5 ± 0.8; 1.6 ± 0.6; and 1.7 ± 0.9 for the superior medial, superior lateral, and inferior medial points, respectively. The total number of fluoroscopy images performed for the group without grid in the three points studied was 12.1 ± 2.5, and for the group with grid, it was 5.0 ± 1.8.

Table 1 shows the statistical difference between the use or non-use of the safety grid found for all the points investigated. The inferior medial point showed the smallest difference between the use or non-use of the grid.

**Table 1:** Number of radioscopies required to achieve correct needle placement for genicular nerve block with and without the use of the adhesive radiopaque grids.

	Non-use of adhesive radiopaque grids	Use of adhesive radiopaque grids	p
Superior medial	5.4 ± 1.5 (range 3-9)	1.5 ± 0.8 (range 1-4)	< 0.0001
Superior lateral	3.6 ± 1.5 (range 1-6)	1.6 ± 0.6 (range 1-3)	< 0.0001
Inferior medial	3.0 ± 1.3 (range 1-6)	1.7 ± 0.9 (range 1-4)	0.0013
total	12.1 ± 2.5 (range 7-17)	5.0 ± 1.8 (range 3-9)	< 0.0001

## DISCUSSION

The main finding of this study is that the use of the adhesive radiopaque grids reduces radiation exposure in cases of genicular nerve block. Ionizing radiation is extremely harmful to humans, being able to directly and cumulatively generate irreversible damage to DNA and the cell membrane even at low doses, especially in cells with a high replication rate, which is the initial event of carcinogenesis. Indirectly, ionizing radiation can also form free radicals that ultimately lead to cell death. ^(^ ^9^ ^),(^ ^10^ Therefore, the use of adhesive radiopaque grids significantly increased the safety of the procedure.

Due to the recurrent use of fluoroscopy in orthopedic procedures, it has been demonstrated that orthopedists have an increased risk of neoplasia/metaplasia compared to other professionals who do not use this tool in their clinical/surgical practice. Among the exposed tissues, the most sensitive to radiation are the eyes, with cataracts being the first sign of chronic radiation exposure; the thyroid, with 85% of papillary carcinomas being induced by radiation; and the hands and gonads, which increase the risk of infertility. ^(^ ^9^ ^),(^ ^10^ ^)^ To minimize the risk of radiation exposure, the International Commission on Radiological Protection (ICRP) established annual radiation dose limits for different tissues. The maximum allowable dose is 20 mSv for the body, 150 mSv for the thyroid and eyes, and 500 mSv for the hands. To prevent exceeding this dose limit, various protective measures are recommended, including the use of personal protective equipment (lead apron, collar, and glasses), which significantly reduces exposure to X-rays (up to 415 times the exposure to the thyroid), and distancing oneself from the fluoroscopy device at the time of image acquisition (being about 2 m away from the image intensifier minimizes radiation exposure).

However, in addition to using personal protective equipment and distancing oneself from the device, reducing the number of fluoroscopies is essential to reducing the amount of radiation to which professionals and patients are exposed. At this point, Malik et al. ^(^ ^11^ demonstrated that the surgeon’s experience led to a significant reduction in the number of fluoroscopies. ^(^ ^10^ ^),(^ ^11^ In addition, important tools have emerged to guide the direction of surgical instruments to reduce the number of images needed to achieve the therapeutic goal.

**Figure 3:**
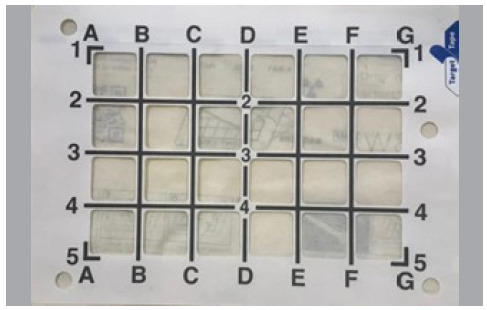
Adhesive radiopaque grid (X-GRID radiopaque adhesive, Target Tape, Canada) used in the study on genicular nerve block.

**Figure 4:**
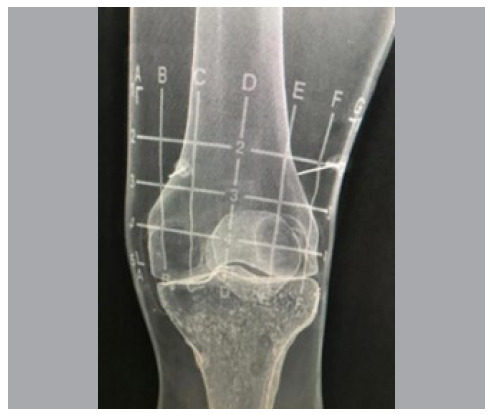
Fluoroscopy image of the model used in the study demonstrating the placement of needles at the superomedial and superolateral points guided by the X-Grid.

In our study, the tool used to guide the needles for the genicular block was the adhesive radiopaque grids. It is an easy-to-use device, with a sterile radiopaque adhesive grid that acts as a guide for needle placement (Figures 3 and 4). Due to the significant anatomical variability among individuals, this tool is of great value for both less experienced professionals and those with extensive experience in the procedure. In the experimental model, an average of 12.1 (± 2.5) fluoroscopies were needed to perform an adequate genicular blockade without using the grid, whereas only an average of 5 (± 1.8) fluoroscopies were needed to perform the same procedure with the grid. This is a statistically significant reduction in the number of images needed, which leads to a reduction in radiation exposure for healthcare professionals and patient. Considering that orthopedic professionals are exposed to radiation in numerous procedures and that radiation has a cumulative effect, the long-term use of adhesive radiopaque grids can mean a significant reduction in the risk of ionizing radiation side effects.

The femoral points showed the greatest difference between using or not using the grid, with the medial femoral point showing the greatest difference. This anatomical positioning is difficult since only the patella and the joint space can be used, especially in patients with thicker thighs. On the other hand, the inferior medial point had the smallest difference between the evaluated points, even so with statistical significance. The possibility of using the anterior tibial tuberosity and the medial cortex of the tibia, located more superficially, makes this point slightly easier than the femoral points.

As a study limitation, we can consider the cross-sectional design, which allows us to generate strong hypotheses about the reduction in radiation exposure, but does not allow us to evaluate the clinical outcome of greater radiation exposure without the use of the grid. Longitudinal studies with this objective are necessary to corroborate our hypothesis. Another limitation is the use of anatomical models with standard anatomy; the results could be more diverse with or without the use of the grid if clinical trials were conducted, since patients tend to have greater anatomical variability, which would make the use of the grid a more helpful tool for correctly positioning needles in these patients.

## CONCLUSION

The use of adhesive radiopaque grids significantly reduced the number of fluoroscopy images required to perform the genicular nerve block in a statistically significant manner. The use of this device increases the safety of the physician and patient by decreasing the exposure to radioscopy in this procedure.
